# Upper- versus lower-extremity synovial sarcoma: distinct surgical pathways and postoperative morbidity in a 24-year cohort

**DOI:** 10.1007/s00432-026-06619-1

**Published:** 2026-09-18

**Authors:** Pia Weskamp, Marius Drysch, Alexander Fiedler, Sonja Verena Schmidt, Felix Reinkemeier, Yonca Steubing, Marcus Lehnhardt, Mehran Dadras, Flemming Puscz, Christoph Wallner

**Affiliations:** 1 Department of Plastic and Reconstructive Surgery, BG University Hospital Bergmannsheil, Ruhr University Bochum, Bürkle-de-la-Camp-Platz 1, 44789 Bochum, Germany; 2 Department of Plastic and Reconstructive Surgery, Agaplesion Diakonieklinikum Hamburg, Hohe Weide, Hamburg, Germany

**Keywords:** Synovial sarcoma, Extremity, Surgical margins, Re-excision, Postoperative complications, Metastasis-free survival

## Abstract

**Purpose:**

Synovial sarcoma is a rare, late-relapsing soft-tissue sarcoma of the extremities. Whether anatomical location shapes the surgical treatment pathway, rather than prognosis alone, is unclear. We compared upper-extremity (UE) and lower-extremity (LE) tumors with respect to margin status, re-excision, reconstruction, morbidity and long-term oncological events.

**Methods:**

Retrospective single-center cohort of 59 consecutive patients with histologically confirmed extremity synovial sarcoma treated between 2000 and 2023. Co-primary endpoints were a microscopically positive margin (R1) at the index resection and any postoperative complication within 8 weeks (Clavien–Dindo). Metastasis-free survival (MFS) and overall survival were exploratory. Fisher exact, Mann–Whitney U, Kaplan–Meier and log-rank methods were used; odds ratios (OR) are UE relative to LE.

**Results:**

Twenty-three tumors were UE and 36 LE. R1 at the index resection was more frequent in UE tumors (14/23 [61%] vs. 5/36 [14%]; OR 9.64, 95% CI 2.73–34.1; *P* < 0.001), as was neoadjuvant therapy (39% vs. 8%; *P* = 0.007). Definitive R0 was achieved in all patients. Any postoperative complication (13% vs. 53%; OR 0.13, 95% CI 0.03–0.53; *P* = 0.002) and major complications (9% vs. 39%; *P* = 0.015) were more frequent after LE surgery. Seven of nine distant metastases occurred in LE tumors, several beyond six years; ten-year MFS was 90% versus 56% (log-rank *P* = 0.095).

**Conclusion:**

Upper- and lower-extremity synovial sarcomas followed distinct surgical pathways and carried distinct morbidity profiles, whereas definitive R0 resection was attained in both. The time-to-event findings are exploratory and require multicenter validation.

**Supplementary Information:**

The online version contains supplementary material available at https://doi.org/10.1007/s00432-026-06619-1.

## Introduction

Synovial sarcoma is a rare, molecularly defined soft-tissue sarcoma characterized by the pathognomonic *SS18::SSX* fusion arising from the t(X;18)(p11;q11) translocation (WHO Classification of Tumours Editorial Board 2020). It predominantly affects adolescents and younger adults, accounts for roughly 5–10% of soft-tissue sarcomas and arises most commonly in the extremities (Ferrari et al. 2004; WHO Classification of Tumours Editorial Board 2020; Gronchi et al. 2021). Although frequently regarded as more chemosensitive than other subtypes, it behaves as an aggressive malignancy with a protracted natural history.

That natural history is defined by a persistent capacity for late local and distant relapse. In a multicenter series with a minimum of ten years of follow-up for survivors, distant metastases occurred at a mean of 5.7 years and as late as 16 years after treatment, and roughly a quarter of patients died of disease beyond ten years, a pattern that mandates prolonged surveillance (Krieg et al. 2011). Established prognostic determinants include tumor size, depth, histological grade, patient age and margin status, whereas the *SS18::SSX* fusion variant is not an independent prognostic factor once grade is accounted for (Trassard et al. 2001; Deshmukh et al. 2004; Guillou et al. 2004). The determinants of occurrence and of relapse in soft-tissue sarcoma overlap only in part, and relapse risk persists long after the index treatment (Weskamp et al. 2022). Anatomical site has itself been linked to outcome, in that distal extremity location has been associated with more favorable survival in localized disease (Deshmukh et al. 2004).

Anatomical site, however, may influence far more than prognosis, in that it constrains the entire surgical treatment pathway. In the hand, wrist and forearm, tumors abut critical nerves, vessels, tendons or joints within narrow compartments, so that a primary wide excision with a generous cuff of normal tissue is frequently not feasible without unacceptable functional loss; anatomical location has been shown to influence the success of limb-sparing surgery in extremity sarcoma (Korah et al. 2012). The same anatomy governs the likelihood of margin involvement at the index procedure, the need for planned re-excision, the selection of neoadjuvant regional treatment such as isolated limb perfusion, the reconstructive requirements for wound closure and the risk of postoperative wound-healing complications, which are well described after lower-limb sarcoma surgery (Cannon et al. 2006). Each of these is a decision point that the treating team must anticipate at presentation.

Although anatomical site has been incorporated into prognostic analyses, its relationship with the complete surgical treatment pathway—from initial margin status and re-excision to reconstruction, postoperative morbidity and late oncological events—has not been systematically characterized. We therefore compared the surgical management, reconstructive requirements, postoperative morbidity and long-term oncological outcomes of upper- and lower-extremity synovial sarcoma treated over more than two decades at a single academic sarcoma center. For the present analysis, margin status at the index resection and postoperative morbidity were defined as the co-primary endpoints; the time-to-event comparisons were treated as exploratory, because the number of metastatic and fatal events in a cohort of this size does not support confirmatory survival inference.

## Materials and methods

### Study design and patients

This retrospective cohort study was conducted at a single academic sarcoma center and is reported in accordance with the Strengthening the Reporting of Observational Studies in Epidemiology (STROBE) statement (Online Resource 2). The institutional sarcoma registry (1060 entries) was screened for all consecutive patients who underwent surgical treatment of histologically confirmed extremity synovial sarcoma between January 2000 and December 2023. Diagnoses were confirmed by specialist sarcoma pathologists according to the prevailing World Health Organization classification (WHO Classification of Tumours Editorial Board 2020), and all cases were discussed at a multidisciplinary tumor board. Tumors of truncal, retroperitoneal or head-and-neck origin were excluded by design, as the study addressed the anatomical divide between upper-extremity (UE) and lower-extremity (LE) disease (Online Resource 1). During the 2000–2023 study period, radiotherapy, systemic and regional treatment, and reconstructive options evolved in accordance with contemporary sarcoma practice. Multidisciplinary case review and definitive R0 resection remained the institutional treatment principles throughout the cohort. These changes occurred gradually and were not encoded as discrete treatment-era exposures in the retrospective registry. The study was conducted in accordance with the Declaration of Helsinki and approved by the ethics committee of the Ruhr University Bochum (Approval No. 20–7126); written informed consent for data collection and analysis was obtained from all patients.

### Data collection and definitions

Clinical data were extracted from the institutional sarcoma database and verified by individual chart review. Anatomical location was classified from the operative site as UE (hand, wrist, forearm, upper arm, shoulder) or LE (foot, ankle, lower leg, thigh, hip). Tumor volume was derived from three-dimensional measurements of the resection specimen. Tumor size was additionally classified by T category and stage according to the 8th edition of the American Joint Committee on Cancer (AJCC) staging system for soft-tissue sarcoma of the trunk and extremities, as recorded in the registry; a reliable continuous maximum tumor diameter was not retrievable for the whole cohort, so size is reported categorically and as volume rather than as a continuous diameter. Tumors were graded using the Fédération Nationale des Centres de Lutte Contre le Cancer (FNCLCC) system, and the full three-tier distribution is reported (Guillou et al. 1997). Reconstructive soft-tissue coverage was defined as coverage beyond primary closure and comprised split-thickness skin grafting, local flap reconstruction, or free tissue transfer. Vascular interposition grafting and functional or motor reconstruction were recorded separately. Because individual patients could undergo more than one reconstructive modality, procedure counts were not mutually exclusive and could exceed the number of patients receiving reconstructive soft-tissue coverage. The treatment context of coverage was categorized from the registry as primary treatment, re-excision, or after neoadjuvant treatment. Prospective reconstructive planning was not captured as a structured registry variable and was therefore not retrospectively inferred.

R1 status was defined as a microscopically positive margin (tumor on ink) at the index resection, irrespective of whether that procedure was performed at the study center or externally. Unplanned excision was defined separately as prior surgical removal outside an oncological pathway, that is without pre-operative imaging and biopsy, and was recorded only for patients with a documented referral history; it was not equated with R1 status at the index resection. Re-excision denoted a planned further resection undertaken to clear margins. Definitive R0 denoted a microscopically tumor-free margin after completion of the first treatment episode, including re-excision where performed; no minimum margin width was required for this classification, and margin width was not quantified in the source records. Operating time denotes the duration of the first procedure performed at the study center.

### Endpoints

The co-primary endpoints were a microscopically positive margin (R1) at the index resection and any postoperative complication occurring within 8 weeks of definitive surgery, graded by the Clavien–Dindo classification. Secondary endpoints were major complication (Clavien–Dindo grade III or higher), the definitive R0 rate, the reconstructive soft-tissue coverage rate and local recurrence. Metastasis-free survival (MFS) and overall survival (OS) were exploratory endpoints. MFS was measured from the primary resection to the first distant metastasis; consistent with an event-specific estimand for distant metastasis, patients without metastasis, including those dying of any cause without prior distant metastasis, were censored at last follow-up or death, and this convention is applied consistently throughout. For OS, the event was death from any cause. Disease-specific death was death attributable to synovial sarcoma or its treatment.

### Statistical analysis

Categorical variables were compared using Fisher exact tests and continuous variables using Mann–Whitney U tests. For binary outcomes, odds ratios (OR) with 95% confidence intervals were estimated by the Woolf method, with a Haldane–Anscombe correction of 0.5 where a zero cell was present, and were oriented consistently as UE relative to LE, so that an OR greater than 1 indicates an outcome more frequent in the upper extremity. Because the reported confidence intervals are asymptotic Woolf intervals whereas the *P* values derive from the Fisher exact test, interval bounds and significance thresholds are not exactly concordant in sparse strata; the exact test result takes precedence in interpretation. Survival was estimated by the Kaplan–Meier method and compared by log-rank testing, with months as the unit of time. Denominators for incompletely documented variables (unplanned excision, adjuvant radiotherapy) are reported explicitly as evaluable cases.

**Fig. 1:**
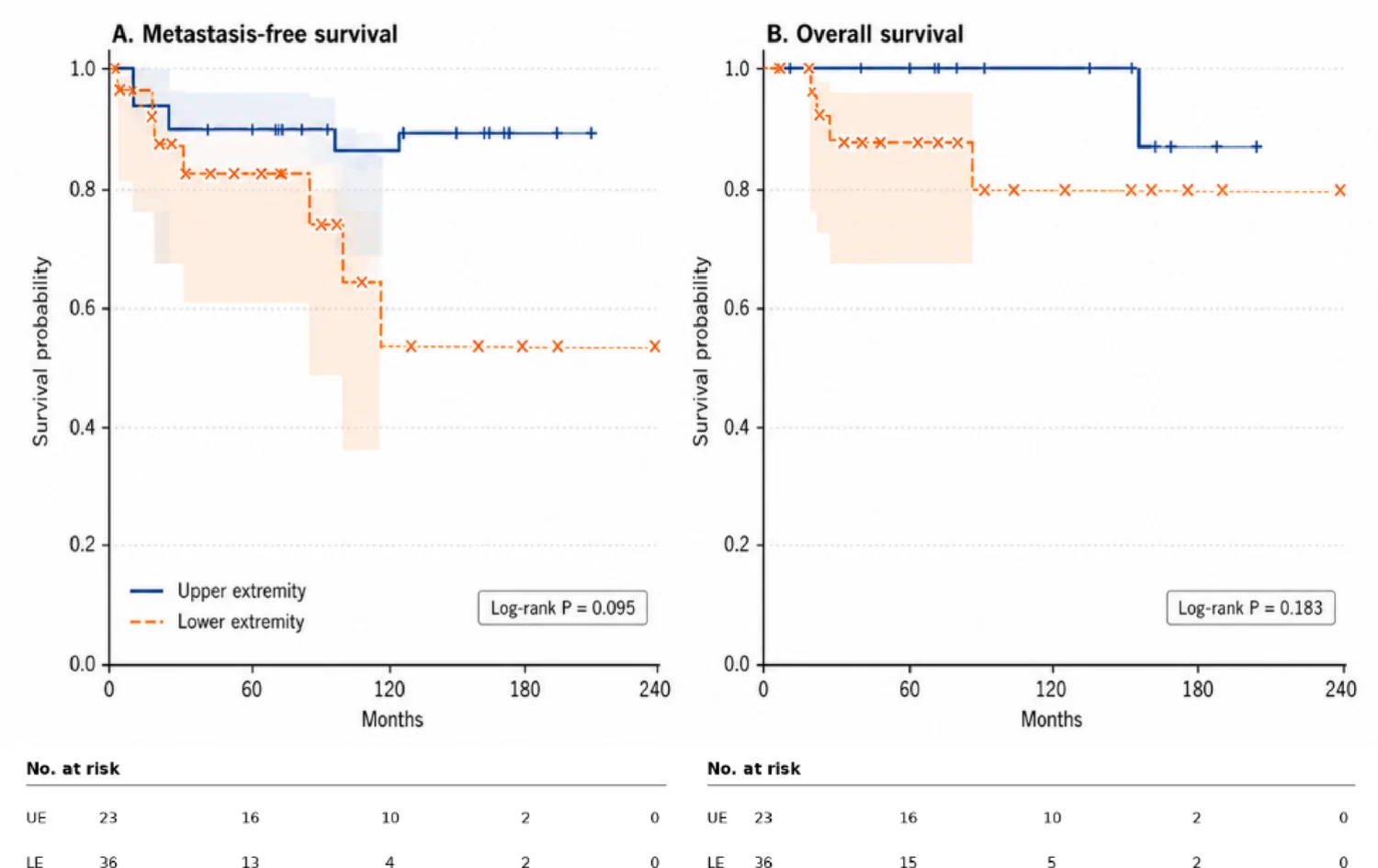
Exploratory time-to-event outcomes in extremity synovial sarcoma. Kaplan–Meier estimates for **a** metastasis-free survival (MFS) and **b** overall survival (OS) in upper-extremity tumors (UE, n = 23; solid navy) and lower-extremity tumors (LE, n = 36; dashed orange). Tick marks denote censoring, shaded bands are 95% confidence intervals, and numbers at risk are shown at 0, 60, 120, 180, and 240 months beneath each panel. MFS diverged beyond 60 months, reflecting late LE distant metastases at 77 to 104 months (log-rank *P* = 0.095); for OS the log-rank P was 0.183, with four of five deaths in the LE group and the single UE death at 154 months. Ten-year estimates rest on few patients at risk beyond 120 months and on unequal median follow-up (UE 85 months, LE 42 months) and are interpreted with caution

A multivariable survival model was not fitted. With nine metastatic events and five deaths, adjustment for multiple potential confounders would have produced unstable estimates, and no adjusted survival estimate is reported. Because deaths without prior distant metastasis were censored in the primary time-to-event analysis, the Kaplan–Meier estimates approximate one minus the cumulative incidence of distant metastasis only under the assumption of independent censoring. A competing-risks sensitivity analysis was therefore performed, in which the cumulative incidence of distant metastasis was estimated by the Aalen–Johansen method with death before distant metastasis as the competing event, and the difference in cumulative incidence at 120 months was tested by a permutation test with 5000 label permutations. No adjustment for multiple comparisons was applied, so *P* values for secondary and exploratory endpoints are descriptive rather than confirmatory. A two-sided *P* < 0.05 was considered statistically significant for the co-primary endpoints. Analyses were performed in Python 3.12 using the lifelines (version 0.29) and SciPy (version 1.14) libraries (Fig. 1).

## Results

### Cohort characteristics

Fifty-nine patients with histologically confirmed extremity synovial sarcoma were identified, of whom 23 (39%) had UE and 36 (61%) had LE tumors (Online Resource 1). No statistically significant differences in the recorded baseline characteristics were observed (Table 1). Median age was 39 years (range 16–72) in the UE group and 34 years (range 14–75) in the LE group (*P* = 0.35); women accounted for 48% of the UE group and 61% of the LE group (*P* = 0.42). Most tumors were subfascial (78% vs 75%; *P* = 1.00). The FNCLCC grade distribution was comparable between the groups, with G1 in 1 UE and 1 LE tumor, G2 in 17 (74%) and 25 (69%) and G3 in 5 (22%) and 10 (28%) respectively (G3 vs G1 and G2 combined, *P* = 0.76). Stage and T category were likewise comparable: among evaluable cases, 13 of 20 UE and 16 of 36 LE tumors were T1 (≤ 5 cm; *P* = 0.17), and AJCC stage III was assigned to 7 of 20 evaluable UE and 19 of 35 evaluable LE tumors (*P* = 0.26). Median tumor volume was numerically smaller in UE tumors (14 cm3, interquartile range [IQR] 3–47, vs 28 cm3, IQR 4–146; *P* = 0.20). Anatomically, UE tumors were concentrated in the hand (n = 11) and forearm (n = 11), with one upper-arm tumor; no wrist or shoulder tumors were separately recorded. LE tumors were located in the thigh (n = 15), lower leg (n = 12), foot (n = 7), and hip (n = 2), with no ankle tumor separately recorded (Online Resource 3). No subsite-level inferential analyses were performed because of the small resulting strata.

**Table 1:** Patient, tumor, and treatment characteristics by anatomical location

Variable	UE (n = 23)	LE (n = 36)	OR (95% CI)^a^	*P*
*Demographics*				
Age, years—median (range)	39 (16–72)	34 (14–75)	–	0.35
Female sex	11 (48)	22 (61)	–	0.42
*Tumor characteristics*				
Tumor volume, cm^3^—median (IQR)	14 (3–47)	28 (4–146)	–	0.20
Subfascial (deep)	18 (78)	27 (75)	–	1.00
FNCLCC grade^b^				0.76
G1	1 (4)	1 (3)		
G2	17 (74)	25 (69)		
G3	5 (22)	10 (28)		
T category (AJCC 8th edition)^c^				0.17
T1 (≤ 5 cm)	13 (57)	16 (44)		
T2 (> 5–10 cm)	7 (30)	18 (50)		
T4 (> 15 cm)	0	2 (6)		
Not assessable (T0/Tx)	3 (13)	0		
AJCC stage (8th edition)^d^				0.26
IA	2 (9)	0		
IB	0	1 (3)		
II	11 (48)	15 (42)		
IIIA	7 (30)	17 (47)		
IIIB	0	2 (6)		
Not assessable	3 (13)	1 (3)		
*Initial treatment pathway*				
Unplanned excision^e^	2/14 (14)	15/34 (44)	0.21 (0.04–1.09)	0.09
Initial R1 resection	14 (61)	5 (14)	9.64 (2.73–34.1)	< 0.001
Re-excision performed^f^	14 (61)	5 (14)	9.64 (2.73–34.1)	< 0.001
Neoadjuvant therapy	9 (39)	3 (8)	7.07 (1.66–30.1)	0.007
*Definitive surgery*				
Definitive R0 resection	23 (100)	36 (100)	–	–^g^
Operating time, min—median^h^	116	106	–	0.40
*Reconstruction*				
Reconstructive soft-tissue coverage	8 (35)	12 (33)	1.07 (0.35–3.21)	1.00
Free flap^i^	2	4		
Local flap^i^	6	7		
Split-thickness skin graft^i^	2	1		
Vascular interposition^i^	2	2		
Motor/functional reconstruction^i^	1	1		
*Adjuvant therapy*				
Adjuvant radiotherapy^e^	11/22 (50)	18/30 (60)	0.67 (0.22–2.02)	0.58
Adjuvant chemotherapy	2 (9)	7 (19)	0.39 (0.07–2.09)	0.46
*Postoperative morbidity (≤ 8 weeks)*				
Any complication	3 (13)	19 (53)	0.13 (0.03–0.53)	0.002
Major complication (Clavien–Dindo ≥III)	2 (9)	14 (39)	0.15 (0.03–0.74)	0.015
Length of stay, days—median	7	10	–	–^j^
*Oncological outcomes*				
Local recurrence	4 (17)	5 (14)	1.31 (0.31–5.47)	0.73
Distant metastasis	2 (9)	7 (19)	0.39 (0.07–2.09)	0.46
Disease-specific death	1 (4)	3 (8)	0.50 (0.05–5.12)	1.00
Death, any cause	1 (4)	4 (11)	–	–

Values are n (%) unless stated otherwise. *P* values derive from the Fisher exact test (categorical variables) or the Mann–Whitney U test (continuous variables)

UE, Upper extremity; LE, Lower extremity; OR, Odds ratio; CI, Confidence interval; IQR, Interquartile range; FNCLCC Fédération Nationale des Centres de Lutte Contre le Cancer

^a^OR is UE relative to LE (Woolf method; Haldane–Anscombe correction of 0.5 where a zero cell was present); an OR greater than 1 indicates an outcome more frequent in the upper extremity

^b^Fédération Nationale des Centres de Lutte Contre le Cancer grading system; the *P* value compares G3 with G1 and G2 combined

^c^T categories follow the AJCC 8th-edition definitions for soft-tissue sarcoma of the trunk and extremities (T1 ≤ 5 cm, T2 > 5–10 cm, T3 > 10–15 cm, T4 >15 cm); no T3 tumor was recorded. The *P* value compares T1 with T2 to T4 among evaluable cases

^d^Stage as recorded in the institutional registry. In one upper-extremity case the recorded stage grouping is not fully consistent with the recorded T category and grade; the registry entry was retained without modification. The *P* value compares stage III with stages I and II among evaluable cases

^e^Denominator is the number of patients with documented status

^f^A planned re-excision was undertaken in every patient with an R1 index resection; this row and the preceding row are therefore identical by construction and are not independent observations

^g^No test is reported because the definitive R0 rate was 100% in both groups and no variability exists

^h^Duration of the first procedure performed at the study center, as recorded in the institutional database

^i^Procedure counts are non-mutually exclusive. Two UE split-thickness skin grafts were combined with local flaps. Vascular interposition and motor/functional reconstruction were recorded separately from the binary reconstructive soft-tissue coverage endpoint; modality counts therefore do not sum to the number of patients receiving soft-tissue coverage

^j^Length of stay was not formally compared between the groups

Kaplan–Meier survival estimates are reported in the Results and in Fig. 1, not in this table

### Surgical treatment pathway

**Fig. 2:**
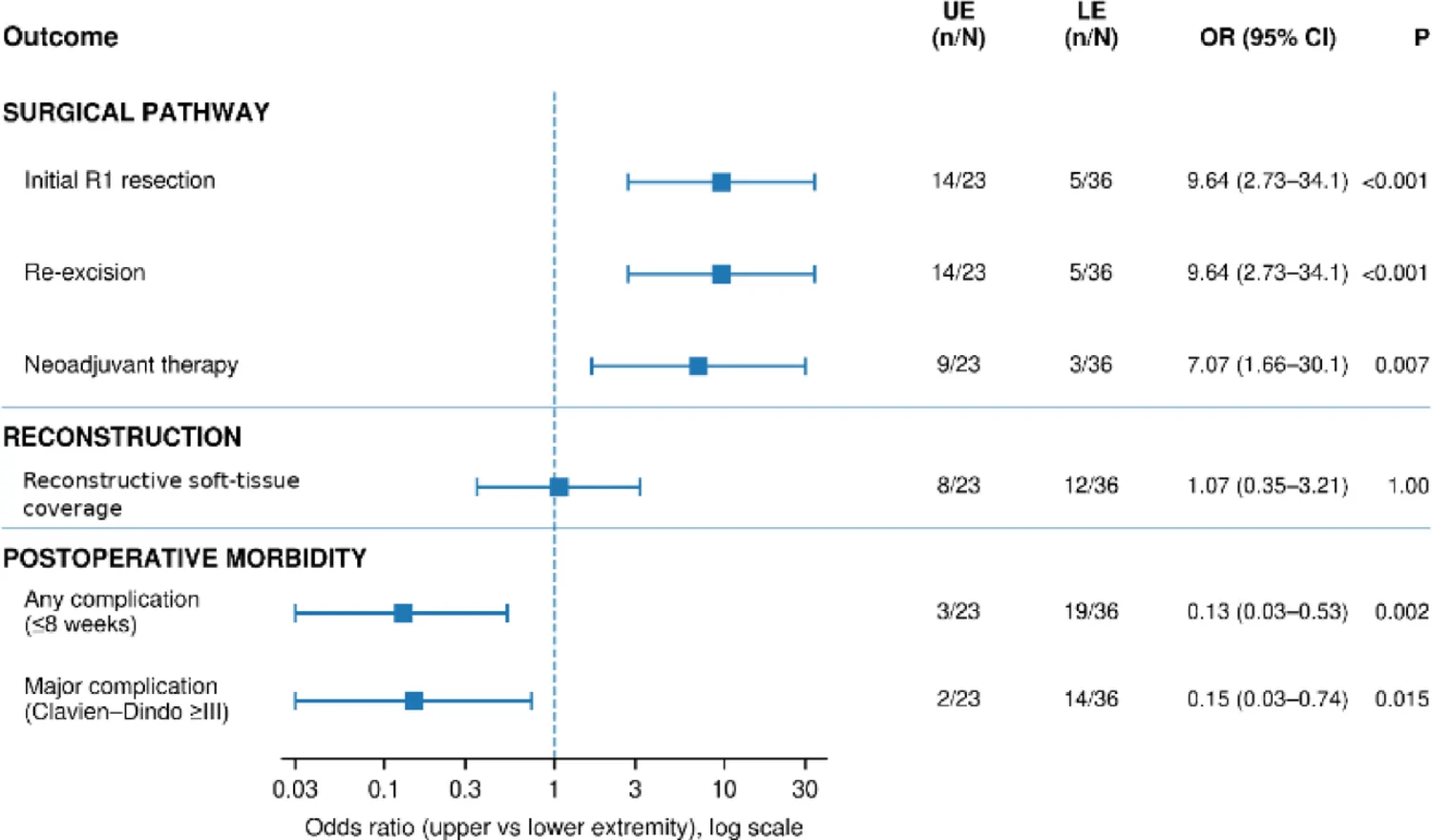
Anatomical location and the surgical treatment pathway. Forest plot of unadjusted odds ratios (UE relative to LE) with 95% confidence intervals and Fisher exact *P* values for the principal treatment-pathway, reconstructive soft-tissue coverage, and postoperative-morbidity outcomes, with events and denominators shown. Relative to the reference line (OR = 1), points to the right indicate outcomes more frequent in the upper extremity and points to the left indicate outcomes more frequent in the lower extremity

The surgical pathways of the two groups differed substantially (Fig. 2). An R1 margin at the index resection was recorded in 14 of 23 UE and 5 of 36 LE tumors (61% vs 14%; OR 9.64, 95% CI 2.73–34.1; *P* < 0.001). A planned re-excision was undertaken in every patient with an R1 index resection, so that the R1 and re-excision proportions are identical by construction; the two rows in Table 1 therefore do not represent independent observations and are reported separately for descriptive completeness only. The higher UE margin-involvement rate was not attributable to prior external surgery, since a documented unplanned excision was in fact less frequent in the UE group (2 of 14 evaluable, 14%) than in the LE group (15 of 34 evaluable, 44%; OR 0.21, 95% CI 0.04–1.09; *P* = 0.09). The available retrospective records did not reliably distinguish deliberately close or marginal oncological dissections from unexpectedly positive microscopic margins. R1 cases were therefore not subclassified by presumed surgical intent, and the higher UE R1 rate should not be interpreted as evidence that microscopically positive margins were planned. Neoadjuvant therapy, which included isolated limb perfusion for selected distal tumors, was administered to 39% of UE and 8% of LE patients (OR 7.07, 95% CI 1.66–30.1; *P* = 0.007). Despite these differences, definitive R0 resection was achieved in all 59 patients, including all 19 who underwent re-excision. Median operating time was similar (116 vs 106 min; *P* = 0.40).

### Reconstruction and postoperative morbidity

The overall rate of reconstructive soft-tissue coverage did not differ significantly between groups (8 of 23, 35%, vs 12 of 36, 33%; OR 1.07, 95% CI 0.35–3.21; *P* = 1.00), although the reconstructive repertoire differed by anatomical context. In the UE group, two free flaps and six local flaps were performed; two of the local flaps were combined with split-thickness skin grafting. Two vascular interposition grafts and one motor or functional reconstruction were also recorded. In the LE group, four free flaps, seven local flaps, one split-thickness skin graft, two vascular interposition grafts, and one motor or functional reconstruction were recorded. Among the 20 patients receiving reconstructive soft-tissue coverage, the recorded treatment context was a re-excision pathway in 13 (6 UE, 7 LE), primary treatment in 6 (1 UE, 5 LE), and after neoadjuvant treatment in 1 UE patient. Prospective reconstructive planning was not systematically recorded. Detailed reconstructive data are provided in Online Resource 3. Adjuvant radiotherapy was given to 11 of 22 evaluable UE patients (50%) and 18 of 30 evaluable LE patients (60%; OR 0.67, 95% CI 0.22–2.02; *P* = 0.58), and adjuvant chemotherapy was infrequent in both groups (9% vs 19%; *P* = 0.46).

**Fig. 3:**
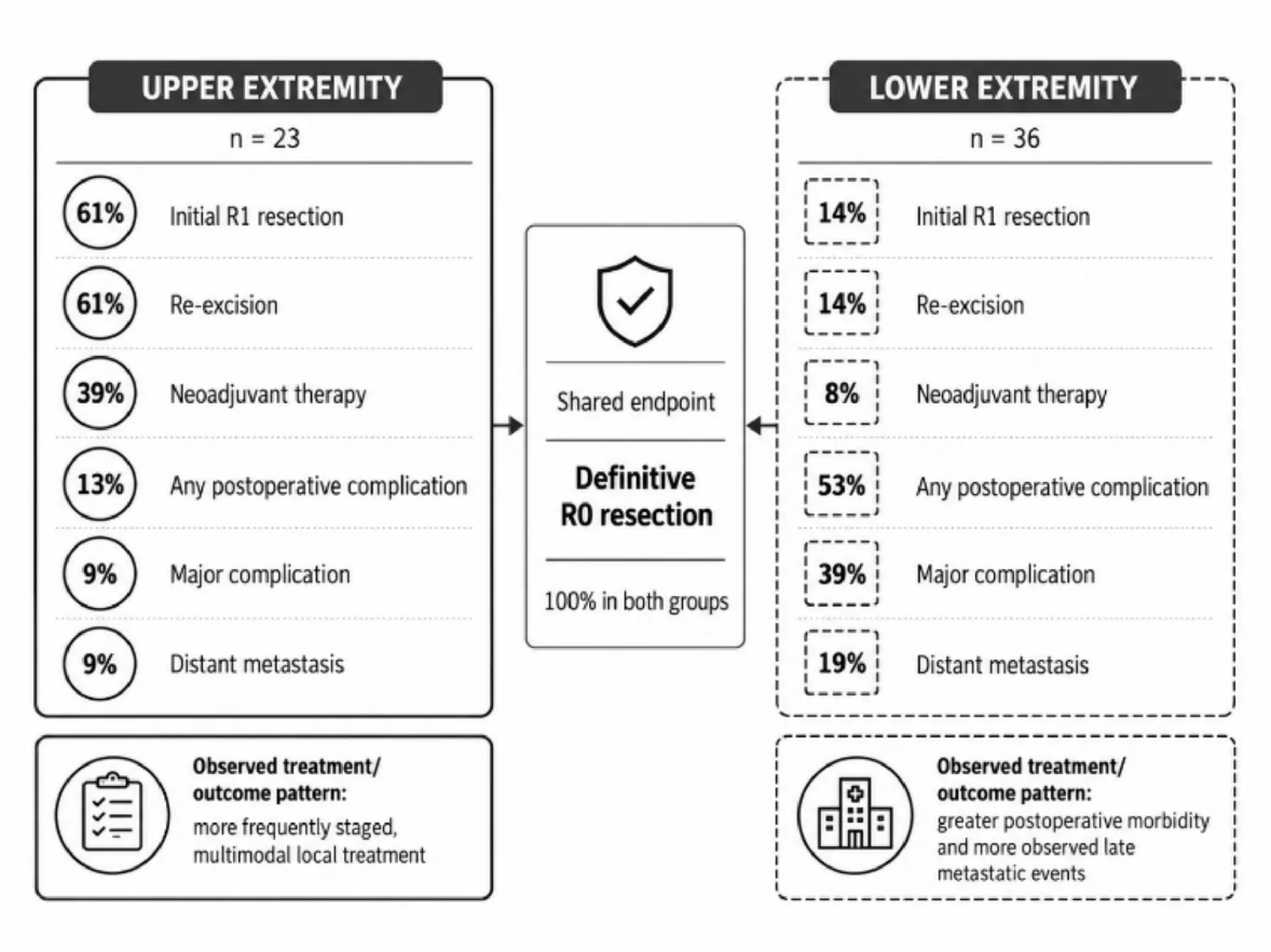
Observed treatment/outcome patterns by anatomical location. Mirrored summary of the observed proportions for the two groups. Upper-extremity tumors (left) more frequently underwent R1 resection at the index procedure, re-excision, and neoadjuvant therapy, with lower postoperative morbidity, whereas lower-extremity tumors (right) showed greater postoperative morbidity and more distant metastatic events. Definitive R0 resection was achieved in 100% of patients in both groups (center). All values are observed proportions within each anatomical group; no causal or biological inference is implied

Postoperative morbidity diverged markedly. Any complication within 8 weeks occurred in 3 of 23 UE and 19 of 36 LE patients (13% vs 53%; OR 0.13, 95% CI 0.03–0.53; *P* = 0.002). Major complications (Clavien–Dindo grade III or higher) occurred in 9% versus 39% (OR 0.15, 95% CI 0.03–0.74; *P* = 0.015). Structured event-level review showed wound infection in 0 UE and 5 LE patients, wound dehiscence in 1 and 2, seroma in 1 and 1, postoperative bleeding or hematoma in 1 and 6, and anemia or transfusion-related events in 0 and 5, respectively. Surgical revision or reoperation was documented in 1 UE and 8 LE patients. These event categories were non-mutually exclusive; a complete descriptive breakdown is provided in Online Resource 3. Median length of stay was 7 days in the UE group and 10 days in the LE group; this variable was not formally compared between groups (Fig. 3).

### Oncological outcomes

At a median follow-up of 85 months in the UE group and 42 months in the LE group, local recurrence occurred in 4 UE (17%) and 5 LE patients (14%; OR 1.31, 95% CI 0.31–5.47; *P* = 0.73). Distant metastasis developed in 2 UE (9%) and 7 LE patients (19%; OR 0.39, 95% CI 0.07–2.09; *P* = 0.46); of the nine metastatic events overall, seven occurred in the LE group. Five deaths occurred overall, one in the UE group and four in the LE group. Disease-specific death occurred in 1 UE patient (4%) and 3 LE patients (8%; *P* = 1.00). The single UE death occurred at 154 months in a patient with synovial sarcoma of the hand who died after local recurrence without prior distant metastasis; this patient was censored for MFS and counted as an event for OS. The remaining LE death was from an intercurrent cause.

### Exploratory time-to-event analysis

Kaplan–Meier curves showed a late divergence in MFS (Fig. 1a). Five-year MFS was 90% in the UE and 84% in the LE group, and ten-year MFS was 90% and 56%, respectively. The observed decline in the LE group corresponded to late distant metastases occurring 77 to 104 months after the primary resection, and the overall difference did not reach statistical significance (log-rank *P* = 0.095). Overall survival at five and ten years was 100% in the UE group and 88% and 81% in the LE group (log-rank *P* = 0.183; Fig. 1b). Because median follow-up was longer in the UE group and few LE patients remained at risk beyond 120 months, the ten-year estimates, and particularly those for the LE group, carry wide confidence intervals and should be interpreted with caution.

Because three patients died without a prior distant metastasis, a competing-risks sensitivity analysis was performed. The Aalen–Johansen cumulative incidence of distant metastasis at ten years was 10.0% (95% CI 1.7–27.2) in the UE group and 41.1% (95% CI 16.0–65.0) in the LE group, while the cumulative incidence of the competing event, death before distant metastasis, was 0 and 8.3% respectively. A permutation test of the between-group difference in cumulative incidence at 120 months yielded *P* = 0.059. The competing-risks estimate for the LE group is accordingly somewhat lower than the corresponding Kaplan–Meier complement (41.1% versus 44%), as expected when a competing event is present, but the direction and the approximate magnitude of the group difference are unchanged.

## Discussion

Upper- and lower-extremity synovial sarcomas followed two distinct surgical pathways. Upper-extremity tumors required staged, multimodal local treatment more often, with margin involvement at the index resection, re-excision and neoadjuvant therapy each several-fold more frequent, yet definitive R0 resection was achieved in every patient. Lower-extremity tumors required fewer staged resections but carried substantially greater postoperative morbidity, and most late metastatic events occurred in this group. Location within the extremity therefore shapes the treatment pathway and its morbidity.

### Anatomical constraints and staged surgical management

The higher rate of margin involvement at the index resection in the UE group, a more than fourfold difference in observed proportions (61% vs 14%; OR 9.64), is consistent with the anatomical constraints of upper-extremity tumor sites, although the retrospective data do not establish the mechanism of margin involvement. A documented unplanned excision was more frequent in the LE group, arguing against prior external excision as the principal explanation for the UE finding. In the hand, wrist and forearm, tumors lie in intimate contact with digital and peripheral nerves, named vessels, tendons and joints, so that a wide cuff of normal tissue at the index operation may be incompatible with limb function. None of this makes a positive margin acceptable. Where it occurs in these locations, however, it should prompt rapid specialist reassessment, multidisciplinary planning and, where feasible, definitive R0 re-excision rather than being treated as an isolated technical failure. The present records could not distinguish deliberately close or marginal dissections from unexpectedly positive microscopic margins, and the observed R1 rate must therefore not be interpreted as planned R1 surgery. This interpretation is supported by the observation that all 19 re-excised patients ultimately achieved R0 and that no evidence of inferior oncological control was observed in the UE group.

Residual tumor after inadequate excision is associated with worse local control and survival across soft-tissue sarcoma (Smolle et al. 2017; Larios et al. 2024), and the present data do not show that re-excision offsets the biological consequences of an initially positive margin. The margin width that matters is in any case entity-specific rather than uniform across histological subtypes, as we have shown for myxofibrosarcoma (Wallner et al. 2026), which is a further reason not to read a uniform R0 rate as uniform margin adequacy. The narrower conclusion is that where anatomical constraints limit the feasibility of a primary wide margin, a staged multidisciplinary pathway incorporating re-excision can still achieve adequate local clearance.

### Definitive R0 as the common endpoint

Definitive R0 resection was nevertheless achieved in every patient, which indicates that margin clearance remains attainable through a staged pathway even in anatomically demanding upper-limb locations, and that the margin result at the index procedure need not determine the final resection status where specialist re-excision is available. This does not imply that the two pathways were oncologically equivalent. Definitive R0 here denotes a microscopically tumor-free margin without a mandated minimum width, so the uniform R0 rate should not be read as uniform margin adequacy in millimetric terms.

### Reconstruction as an enabler of limb preservation

Because the overall rate of reconstructive soft-tissue coverage was almost identical between groups, these data do not support the notion that UE tumors require more soft-tissue coverage. Rather, the reconstructive modalities differed with anatomical context. Vascular interposition and functional reconstruction were recorded separately and further illustrate region-specific reconstructive demands. The broader concept that planned reconstruction can enable limb-preserving oncological resection is supported by the literature (Peat et al. 1994; Tseng et al. 2006; Davidge et al. 2010) but was not directly tested as an outcome determinant in this cohort. Prospective reconstructive planning was not systematically documented, and reconstruction showed no association with survival and should not be presented as improving it.

### Postoperative morbidity after lower-extremity surgery

The most robust association in this cohort is the greater postoperative morbidity after lower-extremity surgery: any complication in 53% versus 13% and major complications in 39% versus 9%. This pattern is consistent with the established literature on limb sarcoma surgery, in which complications after lower-extremity resection are frequent and clinically significant (Cannon et al. 2006). The lower limb presents larger operative fields and greater dead space, a less forgiving soft-tissue envelope and mechanical and vascular conditions that can impair wound healing, and larger resection volume has been associated with wound-healing complications (Geller et al. 2007). Radiotherapy can further increase wound risk: in the randomized comparison of preoperative and postoperative radiotherapy for limb sarcoma, major wound complications occurred in 35% of patients after preoperative radiotherapy, with lower-extremity location and larger tumor size identified as significant risk factors, together with measurable late fibrosis and functional cost (O’Sullivan et al. 2002; Davis et al. 2005). The interval between radiotherapy and surgery, together with multiple patient and tumor factors, further modulates wound risk (Griffin et al. 2015; Slump et al. 2019). The higher morbidity observed in the LE group may therefore reflect a combination of anatomical, tumor-related, and treatment-related factors. These mechanisms were not directly measured in the present study, and the observed association should remain descriptive.

### The late metastatic pattern

Synovial sarcoma is distinguished among soft-tissue sarcomas by a prolonged natural history, with distant metastases occurring well beyond five and even ten years (Krieg et al. 2011). The late-event pattern in the LE group of this cohort, comprising seven of nine metastases with several occurring between 77 and 104 months and corresponding to the late Kaplan–Meier divergence, is consistent with that biology. This carries two implications for practice. A pathway requiring fewer staged resections, or an initially margin-negative resection, must not be equated with long-term oncological safety, since the LE group had the lower rate of margin involvement at the index resection yet the greater number of observed late metastatic events. The divergence emerging beyond five years also reinforces the importance of prolonged surveillance throughout this disease. We do not interpret these data as evidence that lower-extremity location independently predicts metastasis, and we do not propose a separate surveillance protocol confined to lower-extremity tumors; rather, the observed pattern underscores the value of extended follow-up in synovial sarcoma generally and merits external validation.

### Interpretation of the anatomical divide

Several explanations are plausible and they are not mutually exclusive. Distal upper-extremity masses may become clinically apparent earlier, contributing to the smaller UE tumor volumes observed here; smaller size is among the strongest favorable prognostic factors in synovial sarcoma, and distal location has itself been linked to better survival (Deshmukh et al. 2004). More frequent multimodal local treatment in the UE group, including isolated limb perfusion as a recognized limb-salvage strategy in extremity sarcoma (Deroose et al. 2011), may also contribute, as may differences in compartment anatomy and in the success of limb-sparing surgery by location (Korah et al. 2012). Differences in referral pathways, the unequal duration of follow-up between groups and unmeasured tumor heterogeneity cannot be excluded. Disentangling these contributions requires cohorts large enough to support multivariable or machine-learning approaches, which have been applied to prognostic determinants of surgical management in histologically heterogeneous sarcoma populations (Wallner et al. 2025); the event count here does not permit such modeling. Grade distribution did not differ between groups; because fusion-status and other molecular data were incomplete, we make no claim of molecular asymmetry between UE and LE tumors, and the *SS18::SSX* variant is in any case not an established independent prognostic factor once grade is considered (Guillou et al. 2004).

### Clinical implications

Anatomical location should be considered a planning variable rather than a treatment indication in itself. In the present cohort, UE disease was associated with a greater frequency of re-excision and neoadjuvant treatment, emphasizing the need to anticipate potentially staged local treatment and reconstructive or vascular requirements. Conversely, the substantially higher postoperative morbidity observed after LE surgery identifies wound-related complications as an important component of perioperative counseling and planning. Specific measures such as flap coverage, dead-space management, or radiotherapy sequencing are supported by the broader extremity-sarcoma literature but were not evaluated as interventions in this study and cannot be inferred from the present data. Across both anatomical settings, definitive margin clearance and prolonged oncological surveillance remain established principles of care.

### Strengths and limitations

This study is limited by its retrospective single-center design and by the referral and treatment-selection biases inherent to a tertiary sarcoma center. The cohort, although substantial for so rare an entity, yielded few metastatic and fatal events, which precludes robust multivariable survival modeling; the time-to-event comparisons are consequently hypothesis-generating, and the ten-year estimates rest on small numbers at risk. Follow-up was unequal between the groups. Tumor size was available as specimen volume and as the AJCC T category, but not as a reliable continuous maximum diameter, which limits direct comparison with diameter-based prognostic series; margin width and molecular data were likewise incomplete. The competing-risks sensitivity analysis reduces, but does not remove, the interpretive uncertainty of the time-to-event comparison, since it rests on the same nine events and three competing events. The long study period also encompassed evolution in radiotherapy, systemic and regional treatment, and reconstructive practice. Because these changes were gradual and the cohort small, treatment-era effects could not be separated reliably from anatomy or case mix. Standardized functional and patient-reported outcomes were not captured and no adjustment for multiplicity was applied. Prospective reconstructive planning and the surgical intent underlying an R1 index margin were not available as structured variables. These limitations are substantive, but they do not affect the co-primary findings, which rest on large and internally consistent differences in margin status and postoperative morbidity. The strengths of the study are a histologically homogeneous entity, a specialist sarcoma-center cohort observed over more than two decades, detailed surgical and reconstructive documentation, exact treatment-pathway data and the integration of surgical pathway, morbidity and long-term oncological events within a single anatomical framework. Multicenter validation is warranted.

## Conclusions

Upper- and lower-extremity synovial sarcomas follow distinct surgical pathways. Upper-extremity tumors more often require staged, multimodal local treatment, whereas lower-extremity tumors carry substantially greater postoperative morbidity and, in this cohort, more late metastatic events. Definitive R0 resection remained achievable in both settings. These findings support location-informed treatment planning and reinforce the value of prolonged surveillance in this late-relapsing disease.

## Supplementary Information

Below is the link to the electronic supplementary material.

Supplementary Material 1. Cohort assembly. Screening of the institutional sarcoma registry (1,060 entries, January 2000 to December 2023) yielded 59 surgically treated extremity synovial sarcomas, comprising 23 upper-extremity and 36 lower-extremity tumors, after exclusion of entries not meeting the histological or anatomical eligibility criteria. Detailed per-category exclusion counts were not recorded.

Supplementary Material 2

Supplementary Material 3. Tables S1 and S2: Anatomical subsite distribution and reconstructive details, including treatment context, and descriptive event-level breakdown of postoperative complications.

## Data Availability

The datasets generated and analyzed during the current study are available from the corresponding author on reasonable request, subject to applicable ethical and data-protection requirements.
