# Supplementary material for: Upper- versus lower-extremity synovial sarcoma: distinct surgical pathways and postoperative morbidity in a 24-year cohort
Source: J Cancer Res Clin Oncol. 2026 Sep 18;152(9):181. doi: 10.1007/s00432-026-06619-1 (PMC13589721; doi:10.1007/s00432-026-06619-1)
Supplement: Supplementary file 1 [file 432_2026_6619_MOESM1_ESM.pdf]

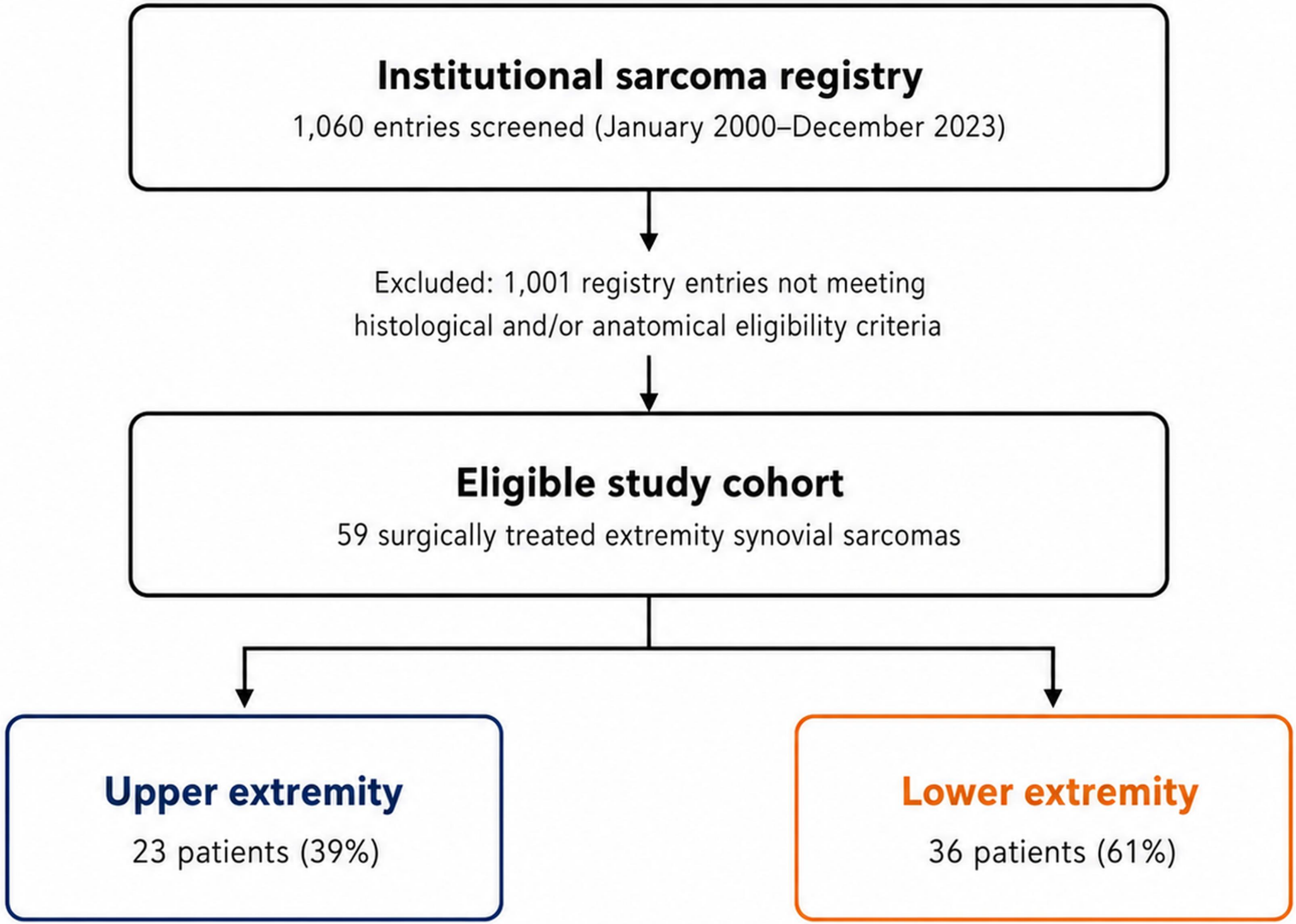

Institutional sarcoma registry
1,060 entries screened (January 2000–December 2023)
Excluded: 1,001 registry entries not meeting
histological and/or anatomical eligibility criteria
Eligible study cohort
59 surgically treated extremity synovial sarcomas
Upper extremity
23 patients (39%)
Lower extremity
36 patients (61%)
