# Supplementary material for: Upper- versus lower-extremity synovial sarcoma: distinct surgical pathways and postoperative morbidity in a 24-year cohort
Source: J Cancer Res Clin Oncol. 2026 Sep 18;152(9):181. doi: 10.1007/s00432-026-06619-1 (PMC13589721; doi:10.1007/s00432-026-06619-1)
Supplement: Supplementary file 2 [file 432_2026_6619_MOESM2_ESM.pdf]

Online Resource 2

STROBE checklist for cohort studies, mapped to the corresponding section of the manuscript

Manuscript: Upper- versus lower-extremity synovial sarcoma: distinct surgical pathways and postoperative morbidity in a 24-year cohort

Item	STROBE recommendation	Location in manuscript
1a	Title and abstract — study design in the title or abstract	Title; Abstract, Methods
1b	Title and abstract — informative and balanced summary	Abstract (structured, 244 words)
2	Background and rationale	Introduction, paragraphs 1–3
3	Objectives, including prespecified hypotheses	Introduction, final paragraph
4	Study design	Materials and methods — Study design and patients
5	Setting, locations, and relevant dates	Materials and methods — Study design and patients
6a	Eligibility criteria, sources, and methods of selection	Study design and patients; Online Resource 1
6b	Matching criteria (not applicable)	Not applicable; unmatched cohort comparison
7	Variables, including outcomes, exposures, and effect modifiers	Data collection and definitions; Endpoints
8	Data sources and methods of assessment	Data collection and definitions
9	Efforts to address potential sources of bias	Statistical analysis; Strengths and limitations
10	Study size	Results — Cohort characteristics (complete registry enumeration; no a priori sample-size calculation)
11	Handling of quantitative variables and groupings	Data collection and definitions; Table 1 footnotes
12a	Statistical methods, including confounding control	Statistical analysis
12b	Methods for subgroups and interactions	Statistical analysis (no subgroup analyses performed)
12c	Handling of missing data	Statistical analysis; evaluable denominators in Table 1
12d	Handling of loss to follow-up	Endpoints; Statistical analysis
12e	Sensitivity analyses	Statistical analysis; Results — competing-risks analysis
13a-c	Participants: numbers at each stage, reasons for non-participation, flow diagram	Results — Cohort characteristics; Online Resource 1
14a	Descriptive data on participants and exposures	Table 1
14b	Number of participants with missing data per variable	Table 1 (evaluable denominators, footnotes c and e)
14c	Follow-up time	Results — Oncological outcomes (median follow-up per group)
15	Outcome data: numbers of outcome events	Results; Table 1
16a	Unadjusted and adjusted estimates with precision	Table 1; Fig. 2 (unadjusted estimates only; see Statistical analysis)
16b	Category boundaries for continuous variables	Table 1 footnotes b and c

Item	STROBE recommendation	Location in manuscript
16c	Translation of relative to absolute risk	Results — observed proportions reported throughout
17	Other analyses performed	Results — Exploratory time-to-event analysis; competing-risks analysis
18	Key results with reference to objectives	Discussion, opening paragraph
19	Limitations, including sources of bias and imprecision	Discussion — Strengths and limitations
20	Overall interpretation	Discussion; Conclusion
21	Generalisability	Discussion — Strengths and limitations; Conclusion
22	Funding	Statements and Declarations — Funding

Section names refer to the headings of the submitted manuscript; page numbers are omitted because they change during typesetting.
