# Supplementary material for: Upper- versus lower-extremity synovial sarcoma: distinct surgical pathways and postoperative morbidity in a 24-year cohort
Source: J Cancer Res Clin Oncol. 2026 Sep 18;152(9):181. doi: 10.1007/s00432-026-06619-1 (PMC13589721; doi:10.1007/s00432-026-06619-1)
Supplement: Supplementary file 3 [file 432_2026_6619_MOESM3_ESM.pdf]

Online Resource 3 - Supplementary Tables

Upper- versus lower-extremity synovial sarcoma: distinct surgical pathways and postoperative morbidity in a 24-year cohort

Supplementary Table S1. Anatomical subsite distribution and reconstructive details

Item	UE (n = 23)	LE (n = 36)
**Anatomical subsite**		
Hand	11	-
Wrist	0	-
Forearm	11	-
Upper arm	1	-
Shoulder	0	-
Foot	-	7
Ankle	-	0
Lower leg	-	12
Thigh	-	15
Hip	-	2
**Reconstructive details**		
Reconstructive soft-tissue coverage, n (%)	8 (35)	12 (33)
Free flap, n	2	4
Local flap, n	6	7
Split-thickness skin graft, n	2	1
Vascular interposition graft, n	2	2
Motor/functional reconstruction, n	1	1
**Treatment context of soft-tissue coverage**		
Primary treatment, n	1	5
Re-excision pathway, n	6	7
After neoadjuvant treatment, n	1	0

Notes. Procedure counts are non-mutually exclusive. Two UE split-thickness skin grafts were combined with local flaps. Vascular interposition and motor/functional reconstruction were recorded separately from the binary soft-tissue coverage endpoint. Prospective reconstructive planning was not captured as a structured registry variable and was not inferred retrospectively. No anatomical subsite-level inferential analyses were performed because of the small resulting strata.

Supplementary Table S2. Detailed postoperative complication profile within 8 weeks

Postoperative event	UE (n = 23)	LE (n = 36)
Any postoperative complication	3 (13)	19 (53)
Major complication (Clavien-Dindo grade >=III)	2 (9)	14 (39)
Wound infection	0	5 (14)
Wound dehiscence	1 (4)	2 (6)
Seroma	1 (4)	1 (3)
Postoperative bleeding/hematoma	1 (4)	6 (17)
Anemia/transfusion-related event	0	5 (14)
Surgical revision/reoperation	1 (4)	8 (22)
Other documented complication	1 (4)	4 (11)

Notes. Values are n (%) using the full anatomical-group denominator. Event categories are non-mutually exclusive and may therefore exceed the number of patients with any complication. “Other documented complication” denotes complication-positive cases not represented in the specified structured event categories. The breakdown is descriptive; no additional inferential tests were performed.
